# Supplementary material for: Deficiency of Adipose Aryl Hydrocarbon Receptor Protects against Diet-Induced Metabolic Dysfunction through Sexually Dimorphic Mechanisms
Source: Cells. 2023 Jun 29;12(13):1748. doi: 10.3390/cells12131748 (PMC10340611; doi:10.3390/cells12131748)
Supplement: Supplementary file 1 [file cells-12-01748-s001.zip › supplementary figlegends.pdf]

Supplementary Figure Legends

Figure S1. Construct of inducible knock-out mouse model using Cre-Loxp system and experimental approach to investigate AhR functionality. Left and Middle Panels: Schematic representation of tamoxifen inducible Cre/loxp mice design and its reporter (tomato) construct to target AhR specifically from adipose tissue driven by adiponectin promoter. Right lane: Experimental plan to investigate AhR loss and its functionality by injecting its agonist BNF (50mg/kg) after tamoxifen injection for two consecutive days. Created by BioRender.com.

Figure S2. Tamoxifen dose dependent tomato expression. (a) Fluorescent microscopy to determine tomato expression of different adipocytes (visceral, subcutaneous, brown: from left to right), when administered 75mg/kg of tamoxifen on HFD. (b) Fluorescent microscopy to determine tomato expression of different adipocytes (visceral, subcutaneous, brown: from left to right), when administered 150mg/kg of tamoxifen on HFD. Abbreviation: VAT, Visceral Adipose Tissue; SAT, Subcutaneous Adipose Tissue; BAT, Brown Adipose Tissue.

Figure S3. CadKO female intake less amount of food and calorie throughout the HFD regimen. Progressive calorie intake and food consumption in male (a) and female (b). $^{**/\#\#}$p<0.01 by 2-way ANOVA with Turkey's post hoc comparison. Abbreviation: HFD, High Fat Diet.

Figure S4. Global and adipose-specific AhR deletion increased mass dependent energy expenditure in females and protects against decreased locomotor activity in both the sexes on HFD. (a-d) Total mass-dependent metabolic rates of AhRKO, CadKO and WT mice were measured at week 13 of HFD diet using the Oxymax Indirect Calorimetry System/Metabolic Cage from Columbus Instruments (CLAMS) for 24 hours. Summary of 24hr VO2 intake (a) and CO2 expelled (b) were used to calculate energy expenditure (c) and respiratory quotient (d). Please refer to Columbus Instruments\Equations for Energy Expenditure and Respiratory Quotient calculation details (http://www.colinst.com/brief.php?id=61). (e) Locomotor activity (n=7-8) under normal light-dark cycle counted for 7 days after 12 week of NCD or HFD using infrared beam interruption sensor. Daily activity was measured by summation of both day and night activity during the period of lights on (7am to 7pm) and lights off (7pm to 7am) respectively and averaged taken from 7 days data. *p<0.05, ****p<0.00001, by 2-way ANOVA with Turkey's post hoc comparison.

Figure S5. CadKO reduces adiposity in female mice. (a-d) Body weight appearance and gross morphology revealing adiposity after 15 weeks of HFD in CadKO (a, c) and WT (b, d) for both the sex.

Figure S6. CadKO reduces adipocytes size in female SAT but not in male. (a) Histological data (H&E staining) obtained on subcutaneous adipose tissue of the CadKO and WT male and female mice after 15 weeks of NCD (n=1) and HFD (n=3-4). Scale bar: 50µm. (b) Adipocyte area frequency distribution plotted for CadKO and WT group in male and female on HFD.

Figure S7. CadKO female increase adiponectin release by white adipose depot. (a) Blood was collected from male and female mice groups (n=8-10), and adiponectin were measured by ELISA. Graph representing adiponectin release normalized to fat mass on HFD**.** (b) Total RNA was isolated from VAT to investigate mRNA levels of adiponectin by real-time PCR on male and female for CadKO (n=4-6) and WT (n=4-6) mice. Each gene was normalized against the amount of housekeeping gene, Actin. Data are

expressed as the ratio of levels in HFD to NCD animals to accentuate changes associated with diet. *$p<0.05$, **$p<0.01$, ****$p<0.0001$ by one-way ANOVA with Turkey's post hoc multiple comparisons test.

Figure S8. CadKO has no effect on non-shivering thermogenesis in BAT. (a) Total RNA was isolated from brown adipose tissue (BAT) to investigate mRNA levels of Ucp1 respectively by real-time PCR on HFD male and female for CadKO (n=4-6) and WT (n=4-6) mice. Each gene was normalized against the amount of housekeeping gene, Actin. (b) Body temperature at room temperature of CadKO and WT after 15 weeks of NCD and HFD for both sexes. (n=3-5) (c) Total RNA was isolated from liver to investigate mRNA levels Fgf21 by real-time PCR on HFD male and female for CadKO (n=5-6) and WT (n=5-6) mice. Each gene was normalized against the amount of housekeeping gene, Actin. (d) Immunofluorescence images of BAT UCP1 expression in CadKO and WT mice after 15 weeks of HFD. Scale bar:100µm.

Figure S9. Liver weight and lipid droplet count on HFD. (a) Liver weight after 15 weeks of HFD in male and female of CadKO (n=3) and WT (n=3). Lipid droplets quantification from the images obtained from H&E. n=3-4. *$p<0.05$, **$p<0.01$, by one-way ANOVA with Turkey's post hoc comparison.
