# Supplementary figures and images for: Deficiency of Adipose Aryl Hydrocarbon Receptor Protects against Diet-Induced Metabolic Dysfunction through Sexually Dimorphic Mechanisms
Source: Cells. 2023 Jun 29;12(13):1748. doi: 10.3390/cells12131748 (PMC10340611; doi:10.3390/cells12131748)

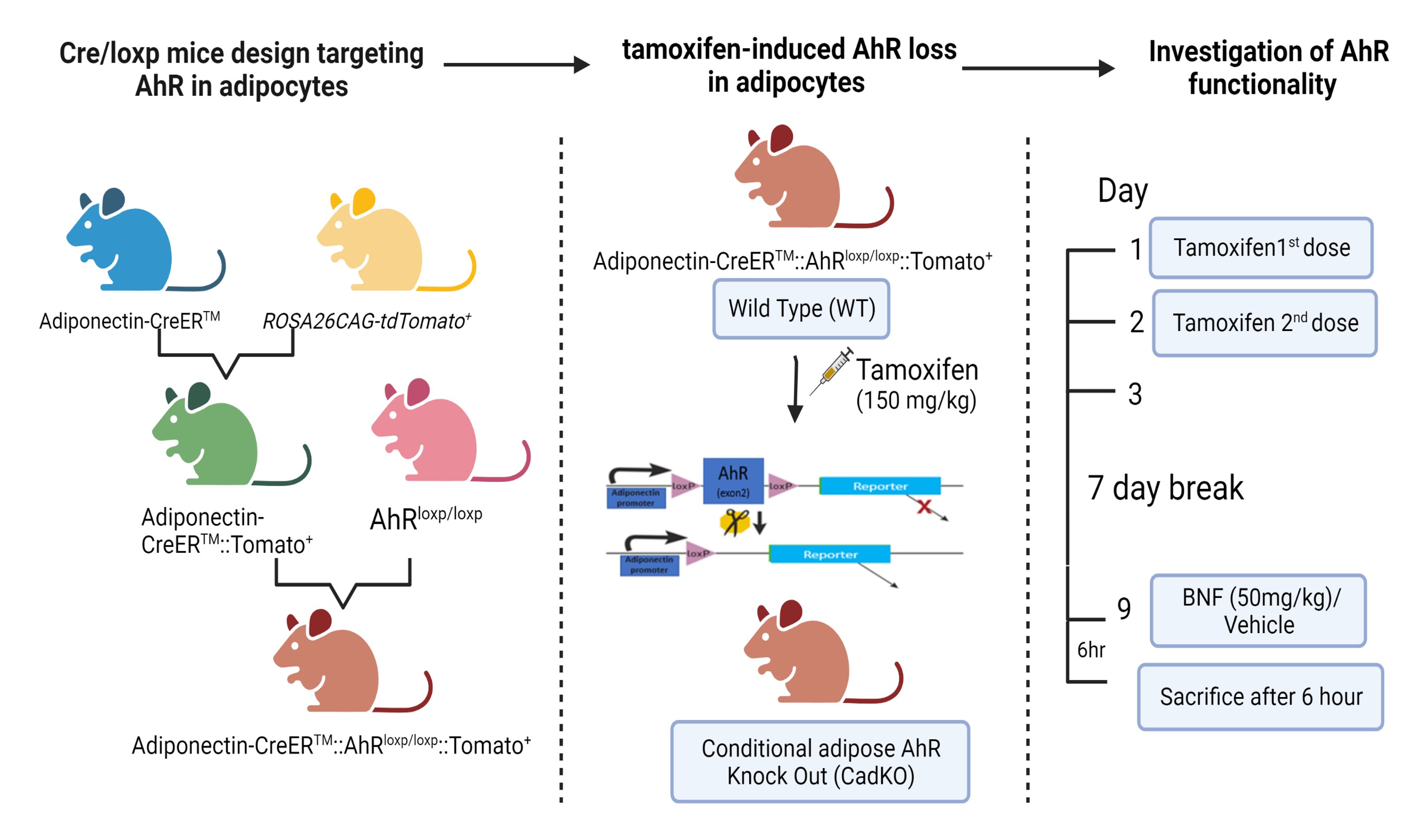

Supplement: Supplementary file 1 [file cells-12-01748-s001.zip › Figure S1.jpg]

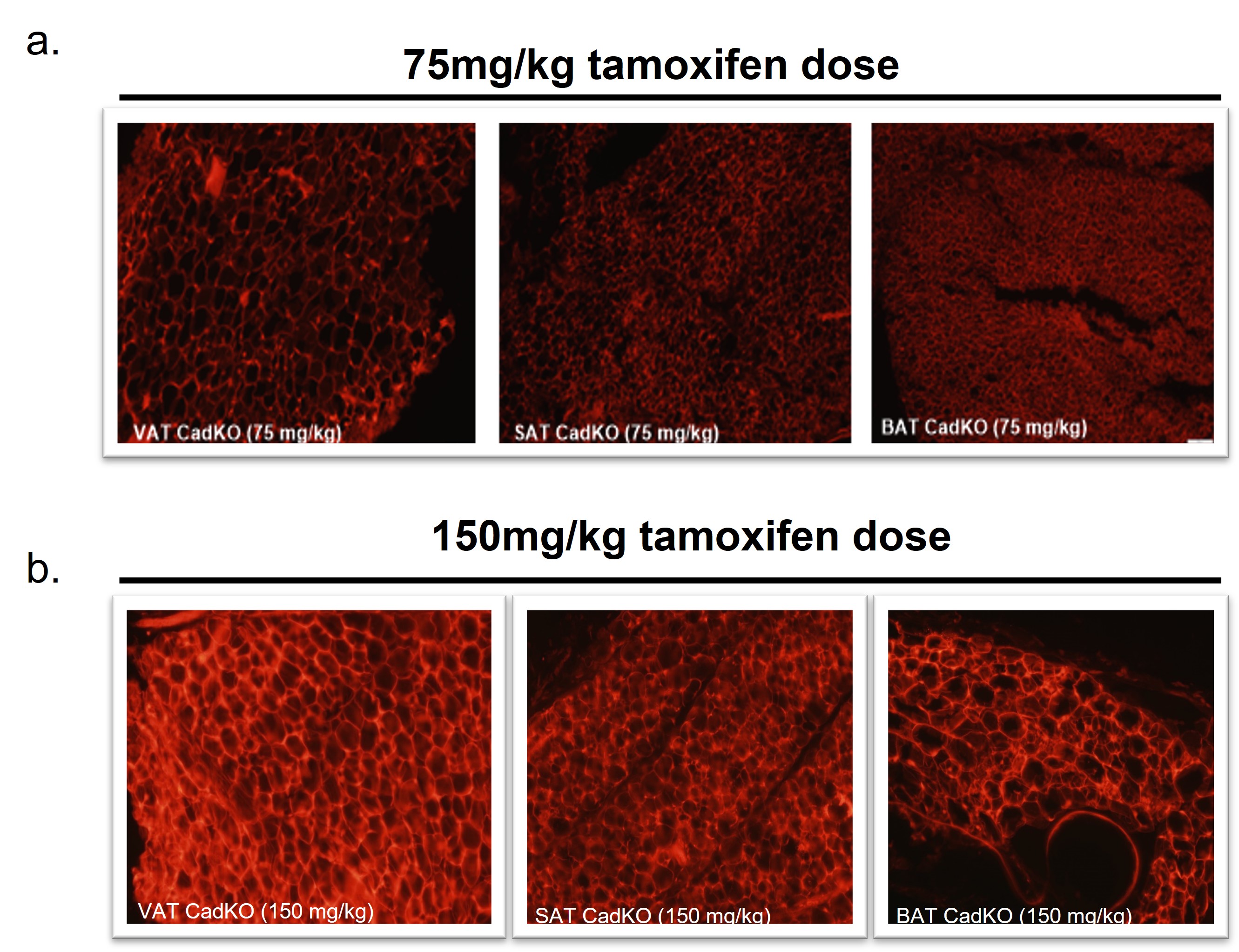

Supplement: Supplementary file 1 [file cells-12-01748-s001.zip › Figure S2.jpg]

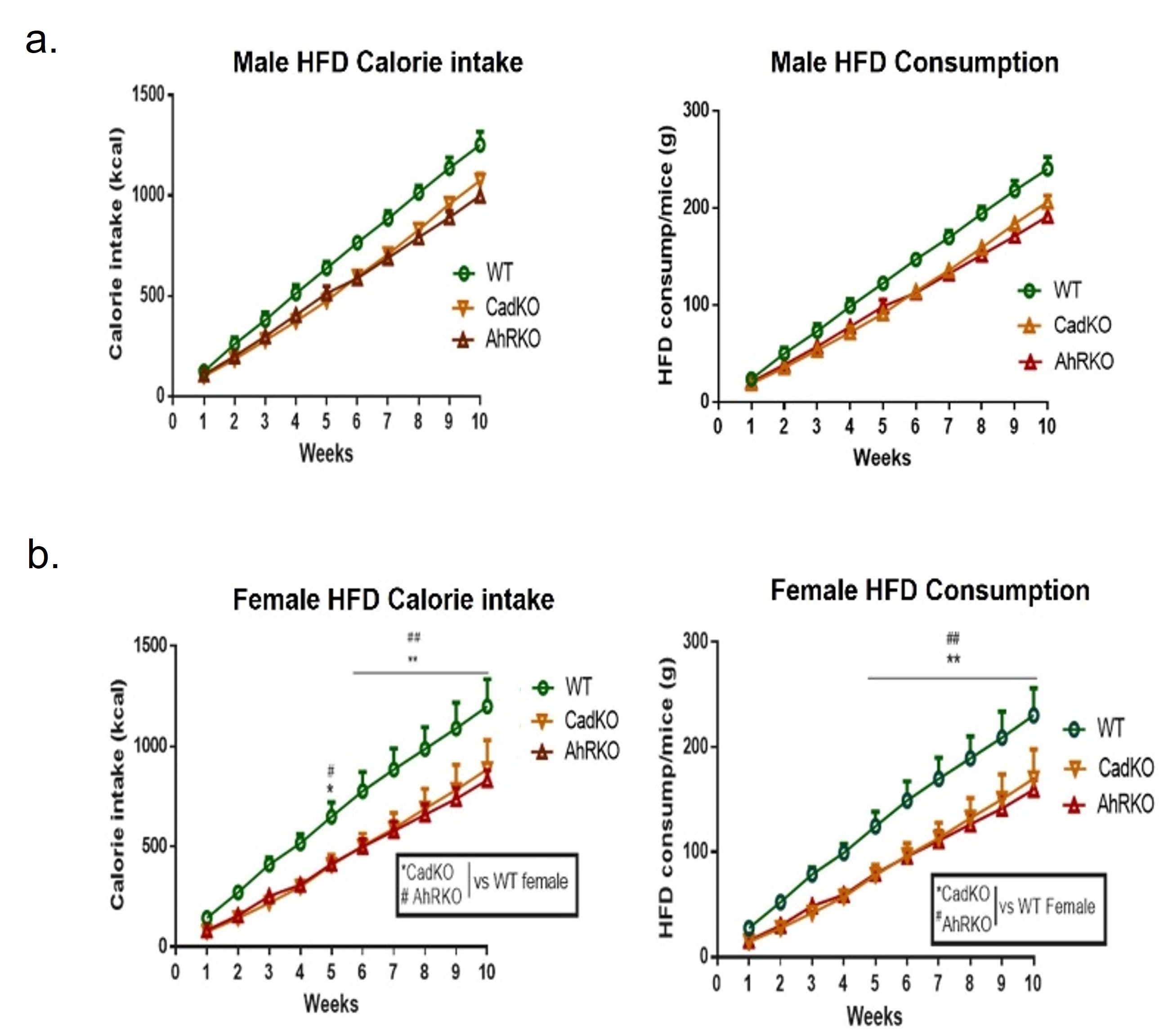

Supplement: Supplementary file 1 [file cells-12-01748-s001.zip › Figure S3.jpg]

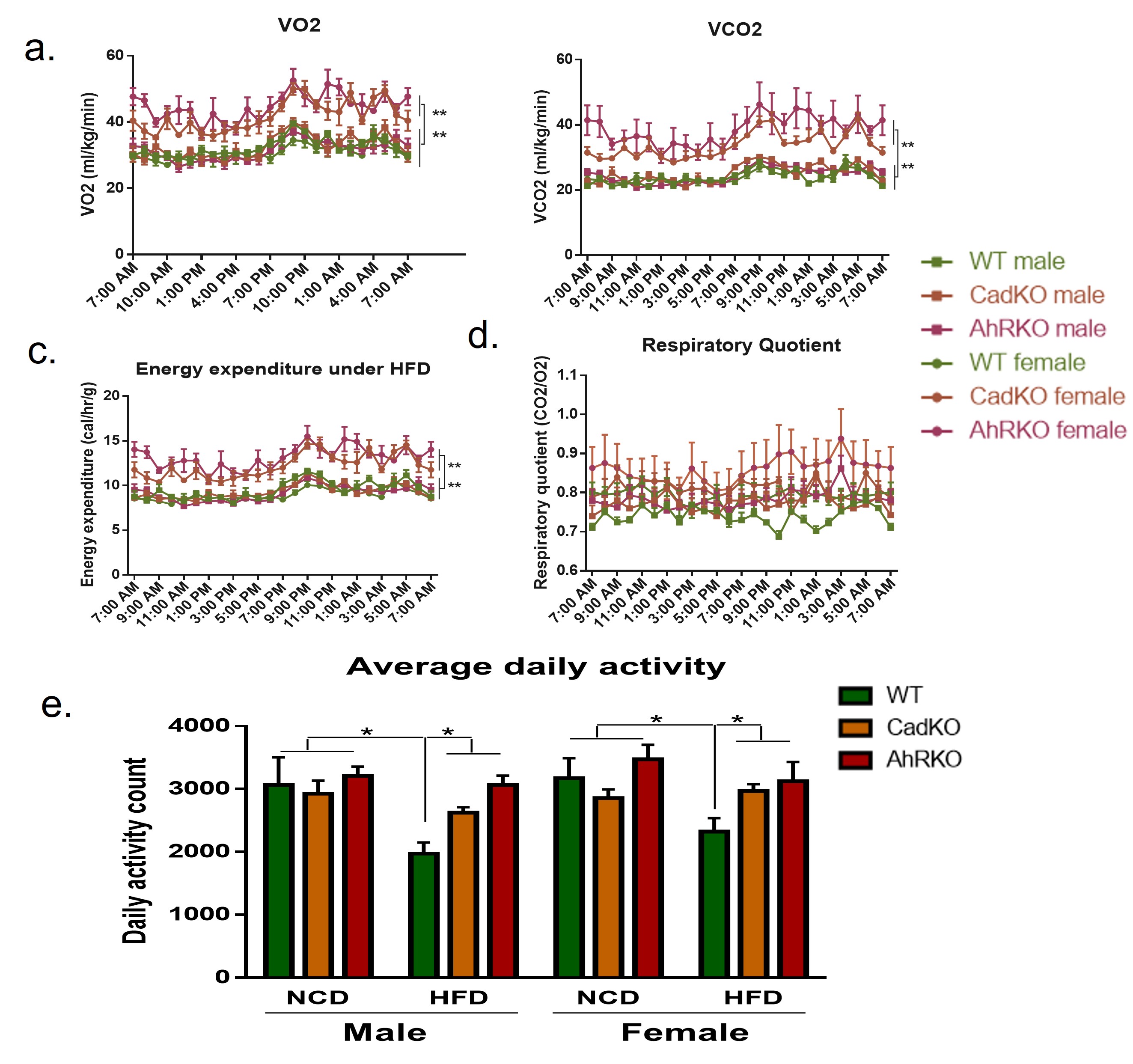

Supplement: Supplementary file 1 [file cells-12-01748-s001.zip › Figure S4.jpg]

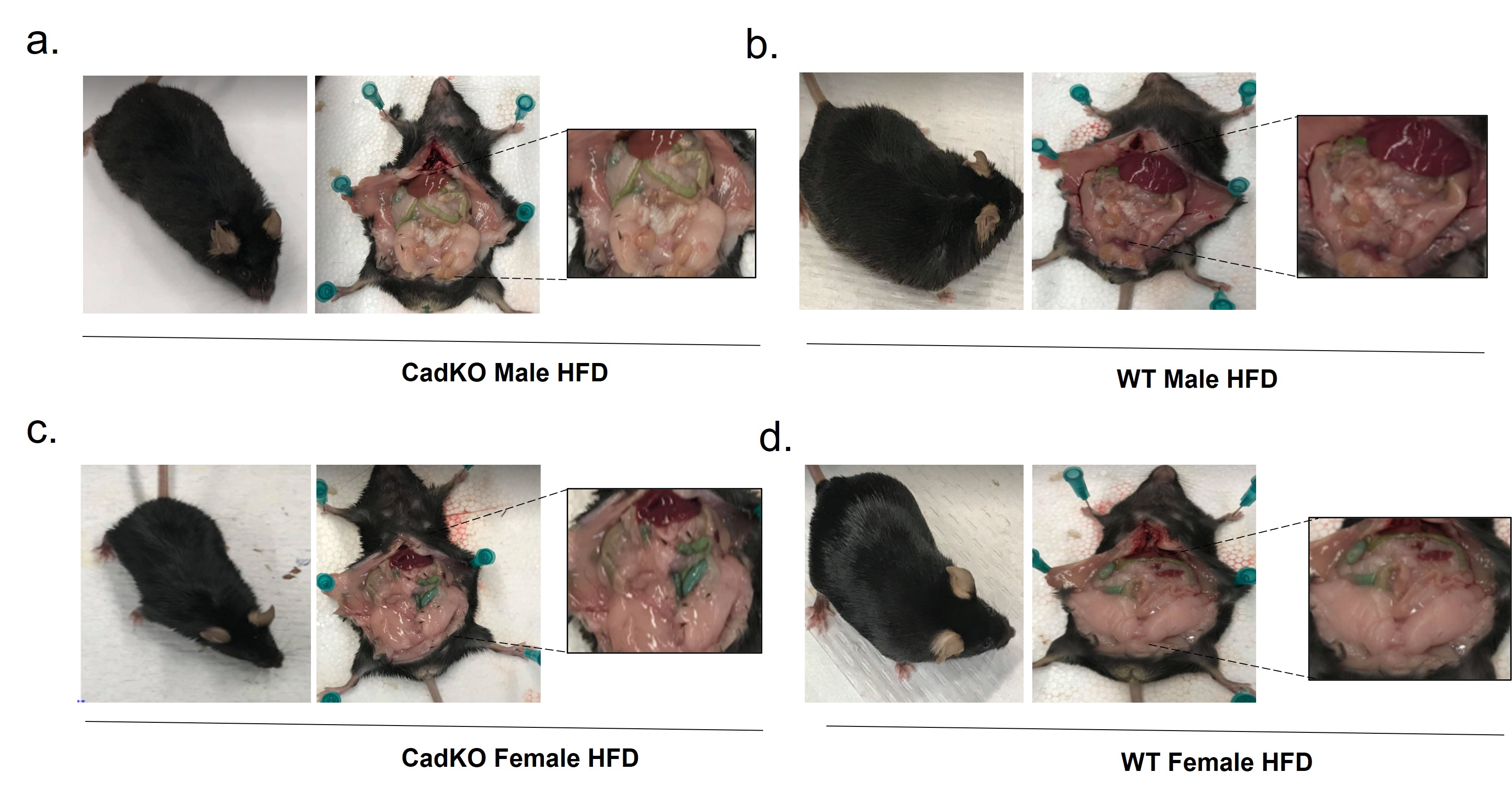

Supplement: Supplementary file 1 [file cells-12-01748-s001.zip › Figure S5.jpg]

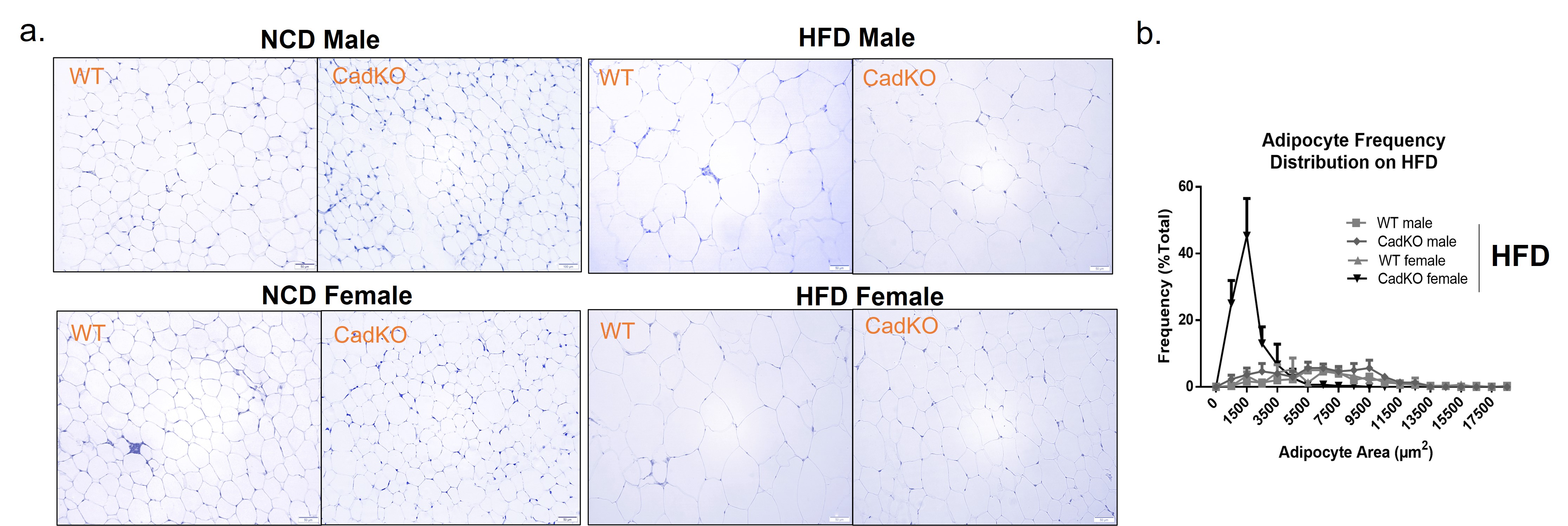

Supplement: Supplementary file 1 [file cells-12-01748-s001.zip › Figure S6.jpg]

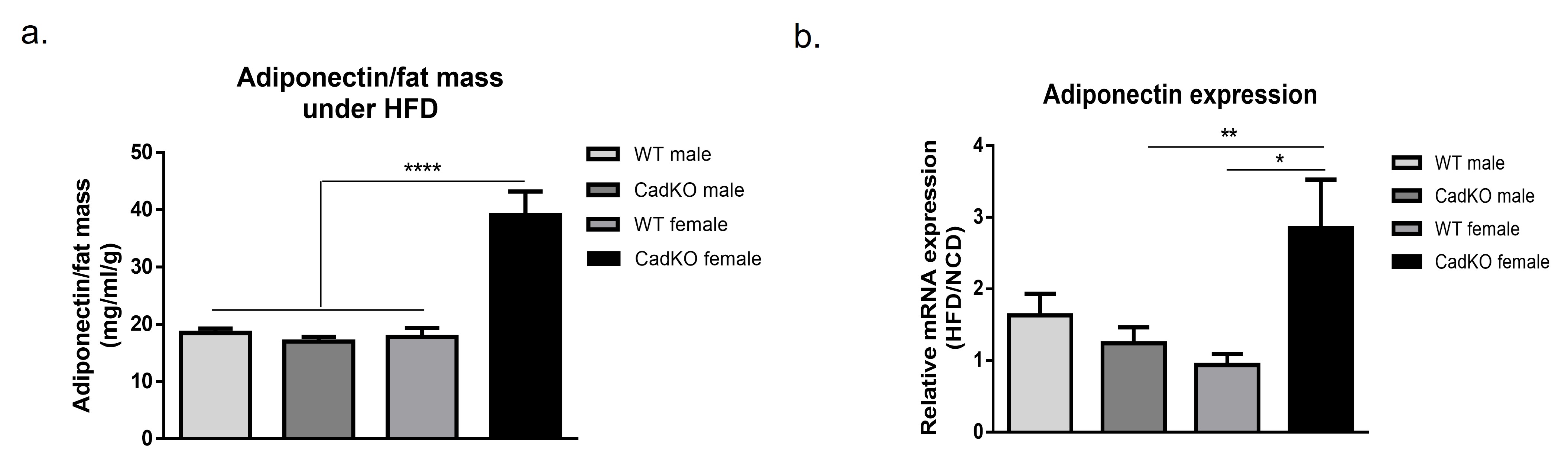

Supplement: Supplementary file 1 [file cells-12-01748-s001.zip › Figure S7.jpg]

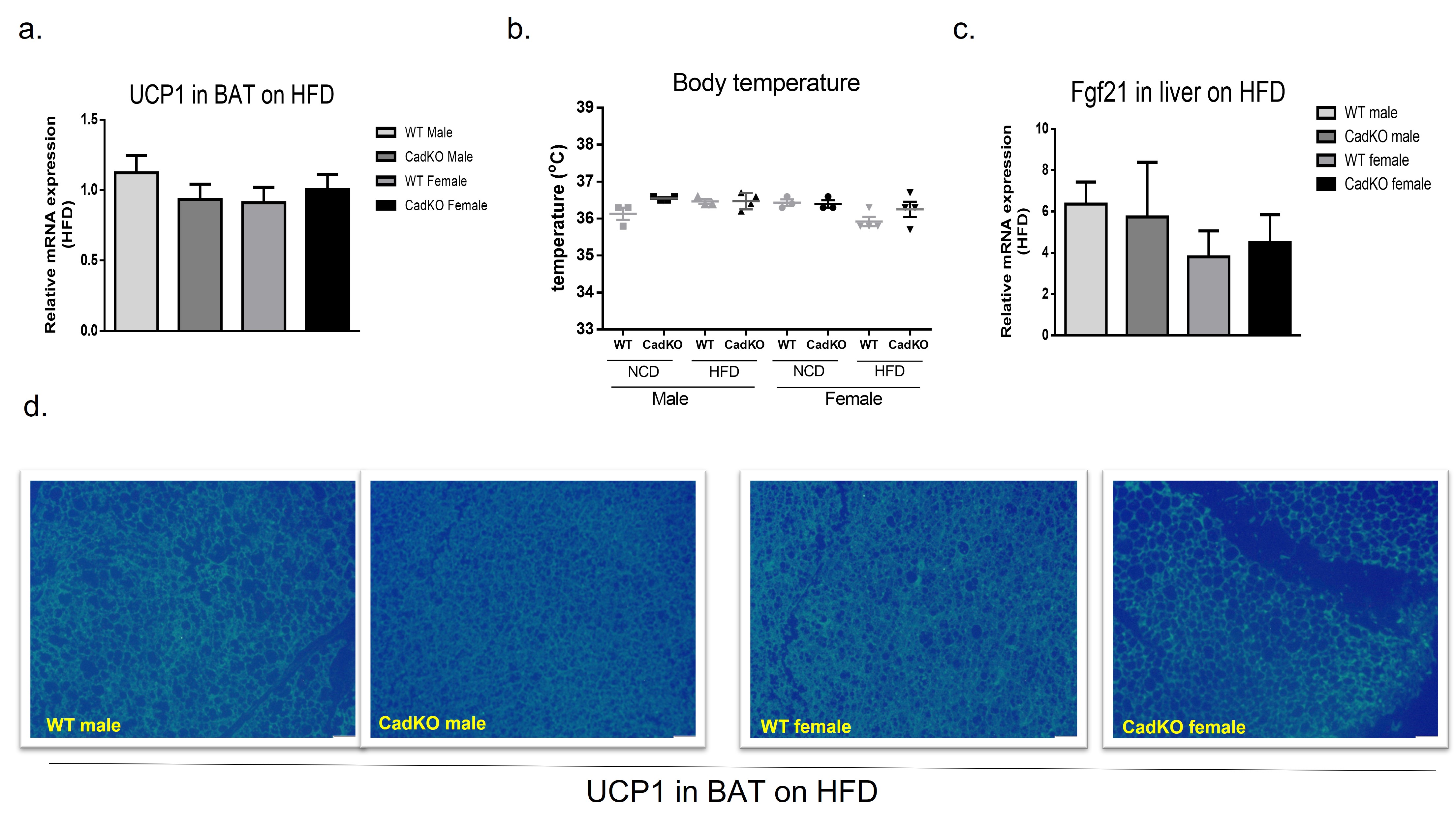

Supplement: Supplementary file 1 [file cells-12-01748-s001.zip › Figure S8.jpg]

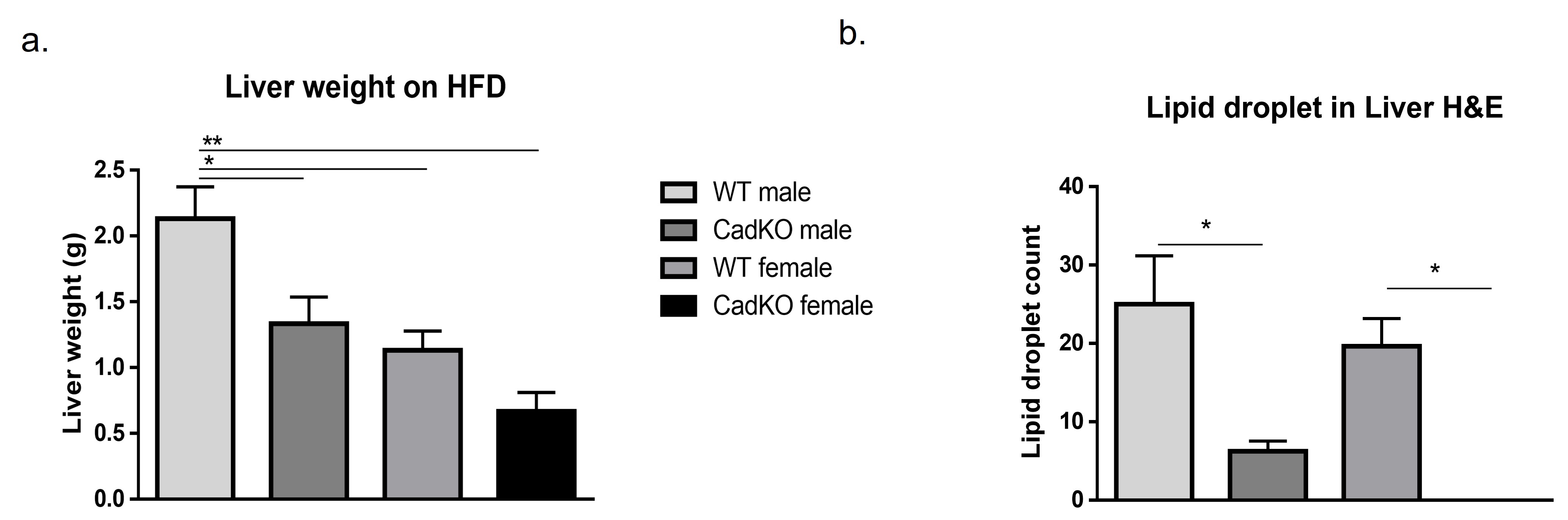

Supplement: Supplementary file 1 [file cells-12-01748-s001.zip › Figure S9.jpg]

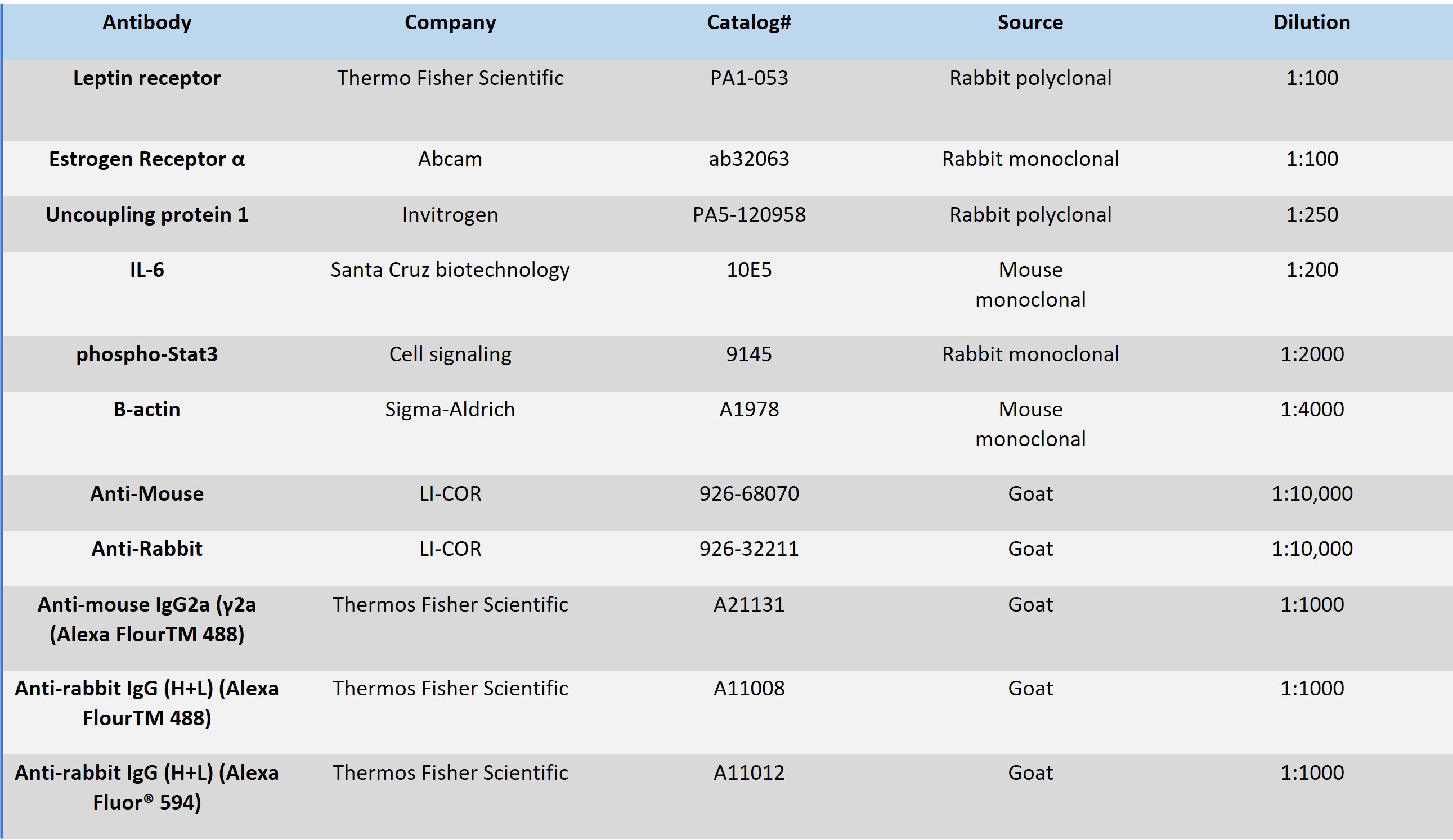

Supplement: Supplementary file 1 [file cells-12-01748-s001.zip › Table S1.jpg]

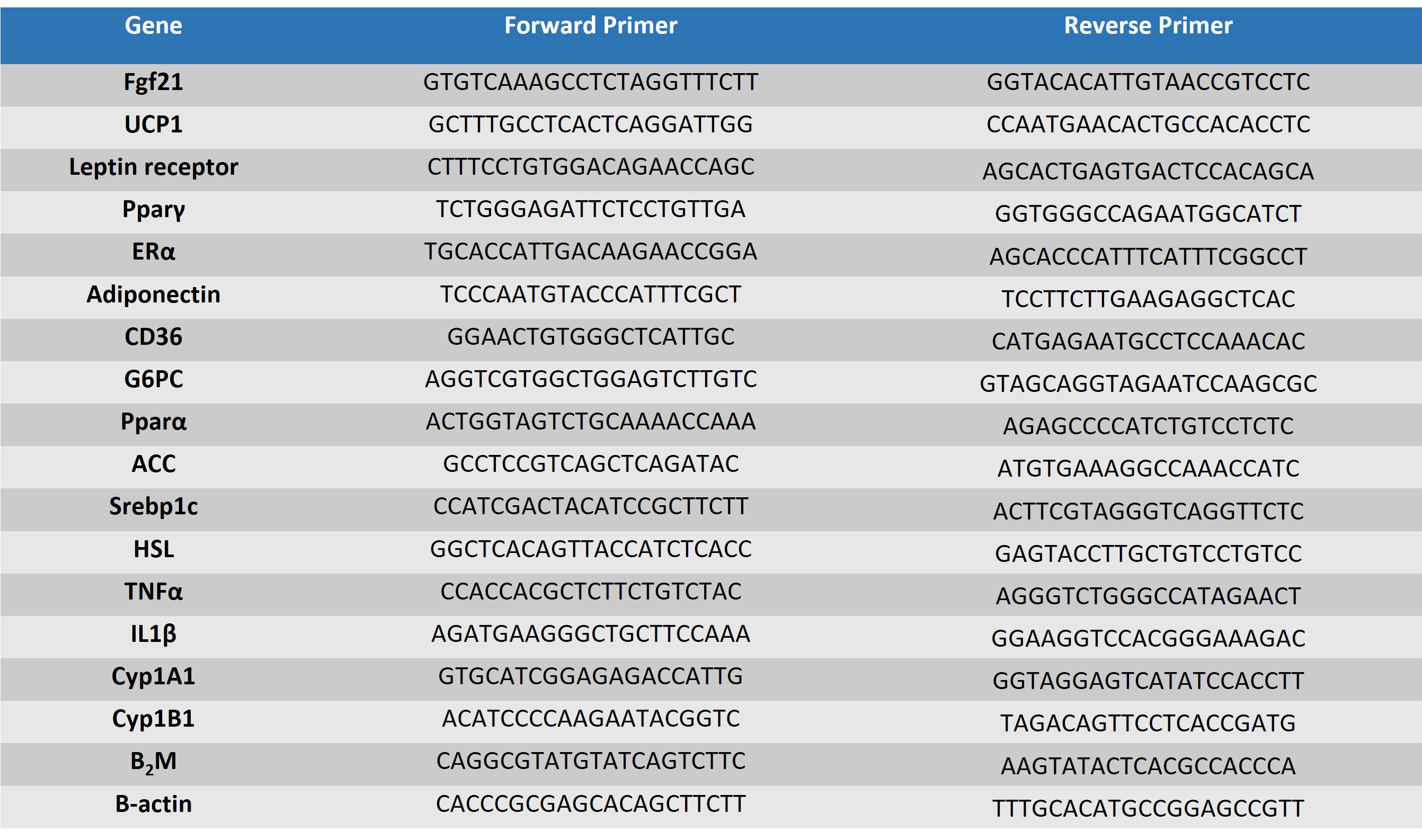

Supplement: Supplementary file 1 [file cells-12-01748-s001.zip › Table S2.jpg]
